# Perceptual and contextual awareness: methodological considerations in the search for the neural correlates of consciousness

**DOI:** 10.3389/fpsyg.2014.00959

**Published:** 2014-08-29

**Authors:** Joaquin Navajas, Hernan G. Rey, Rodrigo Quian Quiroga

**Affiliations:** Centre for Systems Neuroscience, University of LeicesterLeicester, UK

**Keywords:** consciousness, NCCs, contrastive analyses, perceptual awareness, contextual awareness, physical confounds

## Abstract

In the last decades, the neural correlates of consciousness (NCCs) have been explored using both invasive and non-invasive recordings by comparing the brain activity elicited by seen versus unseen visual stimuli (i.e., the contrastive analysis). Here, we review a selection of these studies and discuss a set of considerations to improve the search for the NCCs using the contrastive analysis. In particular, we first argue in favor of implementing paradigms where different perceptual outputs are obtained using identical visual inputs. Second, we propose that the large disagreement in the field -in terms of the dissimilar neural patterns proposed as NCCs- is partially explained by the fact that different studies report the neural correlates of different conscious processes in the brain. More specifically, we distinguish between the *perceptual awareness* of a visual stimulus, associated to a boost in object-selective neural assemblies, and a more elaborate process (*contextual awareness*) that we argue is reflected in the firing of concept neurons in the medial temporal lobe, triggering a rich representation of the context, associations, and memories linked to the specific stimulus.

## INTRODUCTION

When we see a picture of a person, our retinal cells transduce light into electrical signals propagated through the brain, triggering a cascade of neural processes that leads to the conscious percept of the specific person we are looking at. The minimal neuronal mechanisms that are jointly sufficient to elicit a specific conscious percept are known in the literature as the neural correlates of consciousness (NCCs; Crick and Koch, 1990).

In order to empirically manipulate awareness, different methods were developed in the past to render a stimulus invisible despite retinal stimulation (Kim and Blake, 2005). For example, a brief stimulus that is normally visible can become invisible if it is preceded or followed by a second one; a phenomenon called “visual masking” (Enns and Di Lollo, 2000). With “Attentional Blink” (AB), the perception of a salient target presented in rapid visual serial presentation (RSVP) is impaired by the detection of a previous stimulus (Raymond et al., 1992). Similarly, when two clearly different images are sequentially shown separated by a brief blank interval, observers typically fail to detect the change in the images, leading to a manipulation called “Change Blindness” (CB; Simons and Rensink, 2005). These experimental manipulations have in common that the stimulus is transient, i.e., it is presented for a short period of time. However, other techniques allow inducing lack of awareness even with prolonged retinal stimulation. For example, during “Binocular Rivalry” (BR) two disparate images are presented to each eye, causing a sequence of subjective perceptual switches experienced by the observer, suppressing one or the other image despite constant visual stimulation (Blake and Logothetis, 2002). The main downside of BR is that the number of subjective alternations, along with their duration and latencies, are not under experimental control. This issue is absent in a similar technique called “Flash Suppression” (FS) in which one image is presented to one eye, and then is removed from visual awareness by suddenly presenting another image to the other eye (Lansing, 1964; Wolfe, 1984). In the same line, Tsuchiya and Koch (2005) introduced another manipulation, called “Continuous Flash Suppression” (CFS), in which robust and prolonged interocular suppression is achieved by presenting flickering patterns to one eye.

Many previous studies have aimed at finding the NCCs by combining these experimental procedures with different measures of neural activity such as scalp magneto/electro-encephalography (M/EEG; Dehaene et al., 2001; Sergent et al., 2005), functional magnetic resonance imaging (fMRI; Lumer et al., 1998; Portas et al., 2000), intracranial EEG (Fisch et al., 2009; Gaillard et al., 2009), and single-cell recordings in human (Kreiman et al., 2002; Quian Quiroga et al., 2008) and non-human primates (Logothetis, 1998; Macknik and Livingstone, 1998). In general, the methodology undertaken for this line of research is the contrastive analysis, i.e., comparing the neural activity elicited by “seen” versus “unseen” stimuli (Baars, 1993). However, possible drawbacks associated to this empirical approach have been raised in the latest years (e.g., Overgaard, 2004; Aru et al., 2012a). Complementary to these observations, here we discuss a set of considerations to improve the search for the NCCs using the contrastive analysis.

## MANIPULATION OF THE PHYSICAL STIMULI

The first step toward finding the NCCs is to select an experimental method (e.g., backward masking, CFS, etc.) to induce lack of awareness. A simple approach would be to implement this manipulation only for a set of “unseen” trials and contrast the results with a set of “seen” trials, where the manipulation is not used. For example, in the case of CFS, this would imply comparing the neural activity elicited during interocular suppression with a dioptic control in which flickering patterns are absent (Sterzer et al., 2009; Kang et al., 2011; Axelrod et al., 2014). However, the limitation of this approach is that the neural activity induced by the manipulation (e.g., flickering masks in the example of CFS) is absent in the “seen” condition. Therefore, the contrast between “seen” and “unseen” trials could be partially reflecting the processing of different physical stimuli.

An alternative is to use a milder version of the manipulation for the “seen” trials. This can be achieved, for example, by adding different amounts of noise to the stimulus (Jemel et al., 2003) or, in the case of CFS, by changing the contrast of the flickering masks (Kaunitz et al., 2011). But still, differences in low-level features such as luminance, contrast, or spatial frequency can largely modulate brain activity (Scholte et al., 2009). In fact, it has been argued that the ultra-fast detection of faces in natural scenes is partly explained by such features (Honey et al., 2008). One way to reduce these effects is by changing the perceptual ambiguity but controlling for a certain number of low-level variables (Portilla and Simoncelli, 2000; Willenbockel et al., 2010). This strategy ensures that this particular set of variables (e.g., contrast, luminance, and spatial frequency) do not explain differences in the neural activation between “seen” and “unseen” trials. However, the possibility of a hidden low-level variable explaining the differences observed in the neural activations cannot be ruled out.

**FIGURE 1:**
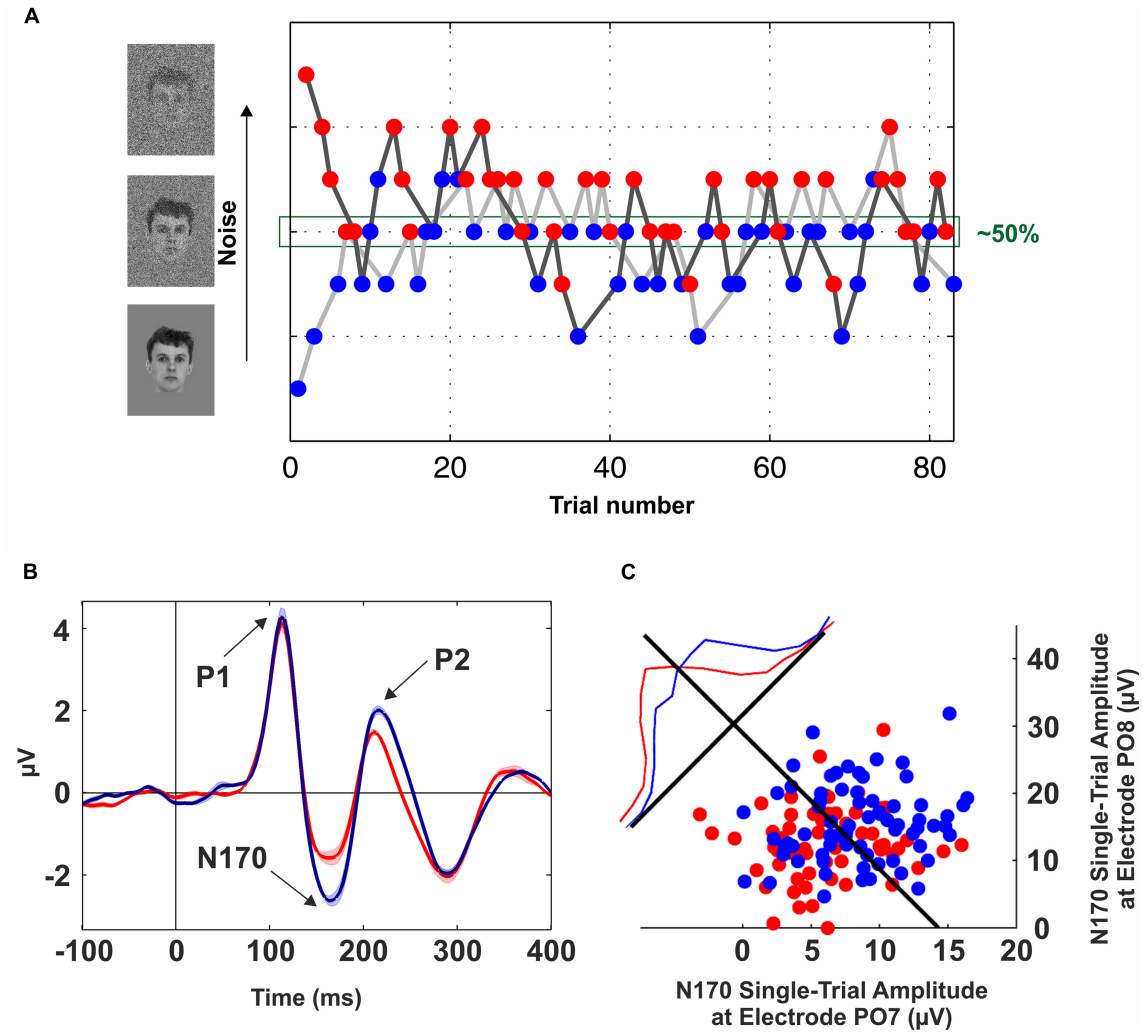
**Neural correlates of conscious face perception. (A)** Brief flashes of faces with different levels of Gaussian noise were presented for 57 ms and combined with backward masking (mask duration: 443 ms). The noise level was tuned on a trial-by-trial basis following a double-staircase procedure, i.e., the noise was increased after a “seen” trial (blue dots) and decreased following an “unseen” trial (red dots). Trials from the upper staircase (dark gray line) and lower staircase (light gray line) were randomly interleaved. This method converges to a noise level of ~50%. Data from one participant of the experiment described in Navajas et al. (2013). **(B)** Grand-average of scalp event-related potentials (ERPs) elicited by “seen” (blue line) and “unseen” (red line) faces obtained with identical visual stimulation. The electrode site (PO8) was in the right occipito-temporal cortex. Three components are observed (P1, N170, and P2); however, the only one that was significantly modulated by conscious perception is the N170. The shaded area around the lines indicates SEM. **(C)** Decoding conscious reports with the single-trial N170 peak amplitude. Blue (Red) dots represent “seen” (“unseen”) trials in two occipito-temporal electrodes (PO7: left hemisphere, PO8: right hemisphere). The blue and red lines show the normalized distributions for “seen” and “unseen” trials projected along the axis perpendicular to the Fisher’s linear discriminant (black line). See Navajas et al. (2013) for further details.

In order to get rid of possible confounds introduced by the physical stimuli, several studies have proposed to compare different perceptual outputs using identical visual stimuli (Sergent et al., 2005; Quian Quiroga et al., 2008; Lamy et al., 2009; Aru et al., 2012b; Navajas et al., 2013). The underlying idea is to implement a manipulation that leads to ~50% recognition performance, and then to contrast the activity elicited by these two sets of trials. The challenge, of course, is to find an experimental manipulation to be at the threshold of perception so that a certain stimulus is equally likely to be recognized or not. For example, Sergent et al. (2005) used the AB paradigm and compared “seen” and “unseen” trials with other set of trials in which the stimulus was absent. Several studies were successful in implementing this approach to uncover the neural basis of visibility (Marois et al., 2004; Sergent et al., 2005). But a challenge when using the AB paradigm is the extremely large variability across individuals (Martens et al., 2006; Willems et al., 2013). In general, due to inter-individual differences (Kanai and Rees, 2011), previous works have proposed to adjust the stimuli ambiguity on a subject-by-subject basis (Fisch et al., 2009; Aru et al., 2012b; Navajas et al., 2013). For example, Navajas et al. (2013) used a modified backward-masking paradigm in which different degrees of zero-mean Gaussian noise were added to the stimuli. Critically, the variance of the noise was tuned across trials following a double-staircase procedure (Cornsweet, 1962; **Figure 1A**). In this way, “seen” and “unseen” trials were extracted from same noise levels, enabling a comparison across different perceptual states but keeping constant the physical stimulation.

Using the contrastive analysis with identical visual stimulation allows ruling out physical effects that can otherwise contaminate the comparison between conditions. Nonetheless, this approach does not exempt the contrastive analysis from other possible confounds (e.g., Aru et al., 2012a). For example, previous efforts have sought to dissociate the NCCs from the effects of attention (Tse et al., 2005; Bahrami et al., 2007), confidence (Sergent et al., 2005; Li et al., 2014), unconscious processing (Lamy et al., 2009; Salti et al., 2012), and introspection (Pitts et al., 2012; Fraessle et al., 2014). Whether the neural correlates of these processes can be entirely disentangled from the NCCs is still matter of extended debate (Lamme, 2003; Block, 2005; Dehaene et al., 2006; Koch and Tsuchiya, 2007; Kouider et al., 2010).

## DIFFERENT CONSCIOUS PROCESSES IN THE BRAIN

### DO DIFFERENT NCCS NECESSARILY CONTRADICT EACH OTHER?

**FIGURE 2:**
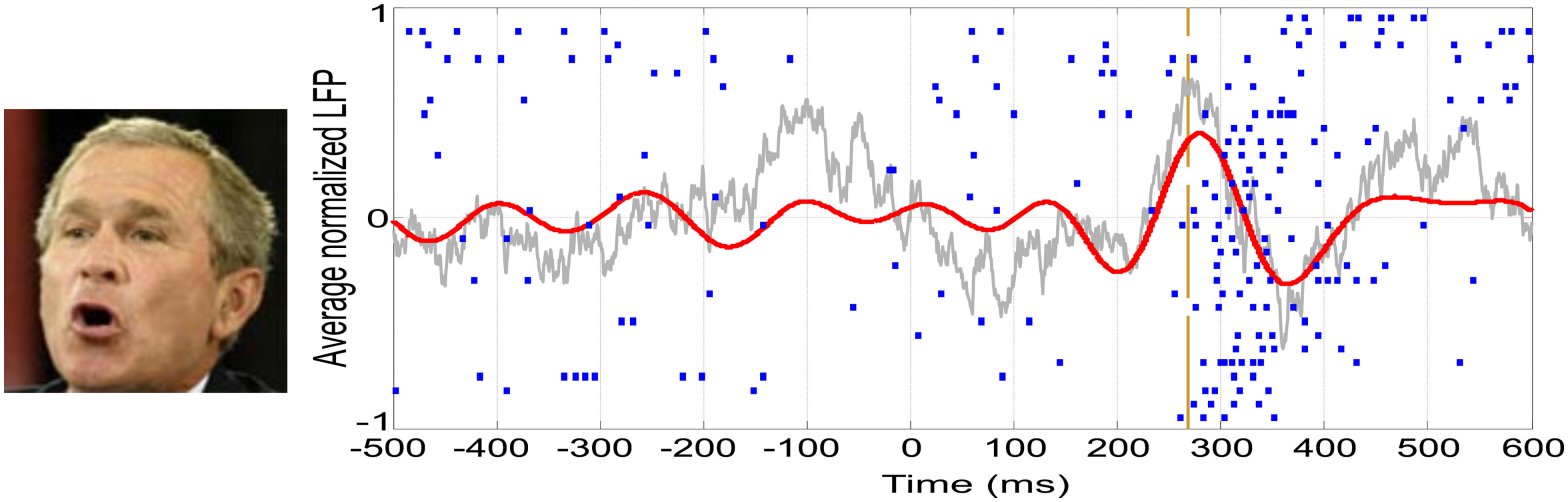
**Single-cell and LFP responses in the human medial temporal lobe.** Example of a neuron in the right hippocampus showing a spiking response to a picture of George W. Bush. Each row in the raster plot is associated to one of the 30 recognized trials. The vertical dashed line represents the onset of the spiking response. The average LFP filtered between 2 and 512 Hz is shown in gray, whereas the one filtered in the theta band (4–8 Hz) is shown in red.

In the last decades, vast empirical and theoretical efforts have been yielded to unravel the NCCs. However, to date, there is little agreement about the areas, timing, and mechanisms involved in eliciting a conscious percept. As an example, our own work has recently provided dissimilar evidence in terms of modulations by awareness occurring at different times and in different areas: (1) An evoked potential measured from the scalp in the OTC peaking at ~170 ms that predicts conscious face perception (Navajas et al., 2013; **Figure 1B**); (2) Single-cell firing at ~300–400 ms of highly selective neurons in the medial temporal lobe (MTL) appearing only upon conscious recognition (Quian Quiroga et al., 2008; **Figure 2**); and (3) A deflection in the local-field potential (LFP) preceding the firing of MTL neurons that is present only in recognized trials (Rey et al., 2014; **Figure 2**). To put together these results into a coherent framework, we propose to distinguish two different neural processes associated with conscious perception, namely, perceptual and full awareness.

### PERCEPTUAL AWARENESS IN OBJECT-SELECTIVE CORTICAL AREAS

The search for the NCCs has posed the problem of finding which of the neural activations along the visual system correlate with conscious perception (Crick and Koch, 1990). In this line, evidence from psychophysical (He and MacLeod, 2001), physiological (Gawne and Martin, 2000), and neuroimaging (Haynes and Rees, 2005) studies supports the notion that the primary visual cortex (V1) is not directly involved in eliciting conscious percepts (Crick and Koch, 1995; Rees et al., 2002). However, alternative views about the role of V1 in visual awareness were also proposed (Tong, 2003). In turn, object-selective responses in the inferotemporal cortex (ITC) have been consistently shown to be modulated by conscious perception (for a review see Logothetis, 1998). Similarly, an evoked potential in the 5 to 15 Hz frequency band at ~170 ms was repeatedly shown to be modulated by conscious perception using the contrastive analysis with identical visual stimulation (Fisch et al., 2009; Navajas et al., 2013; Sandberg et al., 2013; **Figure 1B**). Furthermore, conscious reports about face perception can be decoded at the single-trial level based on the peak amplitude of these evoked responses (**Figure 1C**).

In principle, this set of results showing correlations between awareness and brain activity at 100–200 ms after stimulus onset could be in conflict with theoretical proposals arguing that the NCCs are reflected by “late” (>300 ms) activations (Dehaene and Changeux, 2011). Alternatively, these neural modulations can be regarded as pre-requisites of consciousness (Aru et al., 2012a) occurring after stimulus onset (VanRullen, 2011), thus reflecting a preconscious state (Dehaene et al., 2006). We believe that this evidence supports the existence of a conscious process for visual recognition, namely perceptual awareness, which is linked to a boost in the activity of object-selective neural assemblies in high-level visual areas. However, we do not claim that this is the only conscious process in the brain. Instead, we propose that the role of perceptual awareness is to rapidly recognize visual stimuli and to feed this information to other neural circuits involved in different cognitive functions. Among the subset of processes, we will focus on one occurring in the MTL that is directly involved in memory processes.

### CONTEXTUAL AWARENESS IN THE MEDIAL TEMPORAL LOBE

Patients with pharmacologically intractable epilepsy, implanted with intracranial electrodes for clinical reasons, provide the unique opportunity to record, with the proper setup, single-cell activity from the conscious human brain (for a review see Engel et al., 2005). With these recordings, it was found that neurons in the MTL respond to different images in a remarkably selective and invariant manner. For instance, one neuron fired to seven different pictures of the actress Jennifer Aniston and not to other 80 pictures of other celebrities, animals and places (Quian Quiroga et al., 2005). Moreover, these responses could be triggered not only by pictures but also by the written name of the person and by the name pronounced by a synthetized voice (Quian Quiroga et al., 2009). Altogether, these results support the idea that the function of these neurons is to provide an explicit and abstract representation of the meaning of stimuli, thus being called *concept cells* (Quian Quiroga, 2012).

The latency of the firing of concept neurons is about 300 ms, although parahippocampal cells fire 50–100 ms before (Mormann et al., 2008). This timing is consistent with a set of “late” (>300 ms) activations that were shown to correlate with access to consciousness (Del Cul et al., 2007; Dehaene and Changeux, 2011). In this line, previous research has shown that neurons in the MTL modulate their firing activity with conscious perception (Kreiman et al., 2000, 2002; Reddy et al., 2006; Quian Quiroga et al., 2008). For example, when two incongruent pictures are presented to each eye, the firing of these neurons follows subjective perception (Kreiman et al., 2002). Similarly, in a CB paradigm, Reddy et al. (2006) showed that concept cells do not follow retinal input, as they were only active upon the perception of the changes. Using a backward-masking paradigm, previous research has also shown that concept cells fire only when the subject recognized the stimulus (Quian Quiroga et al., 2008). Remarkably, the responses appear in an all-or-none fashion, even if the visual stimuli were identical – i.e., the same picture at the same duration (Quian Quiroga et al., 2008).

These studies provide critical evidence for asserting that conscious perception is accompanied by these neural responses in the MTL. However, it was argued that this stage of processing might reflect the consequences of conscious recognition (NCC-co), rather than recognition *per se* (Aru et al., 2012a; Quian Quiroga, 2012). This claim is supported by the fact that damage to MTL structures does not impair conscious perception (Kensinger and Corkin, 2000; Postle, 2009). Likewise, here we propose that perceptual awareness precedes the firing of concept cells and is correlated with neuronal firing at 100–200 ms after stimulus onset, probably in the ITC (Logothetis, 1998). Indeed, we believe that a different and more sophisticated conscious process is triggered when this information is propagated to the MTL, activating these highly selective and sparsely firing neurons that represent the meaning of the stimulus for declarative, and particularly episodic, memory functions (Quian Quiroga, 2012). In particular, we support the idea that full awareness of the stimulus is elicited by this sensory-independent conceptual representation.

### FROM RECOGNITION TO CONTEXT: A LINKING MECHANISM?

One of the most intriguing aspects of concept cells is the fact that their mean firing onset is too late (~300 ms) to be explained by direct projections from high-level visual areas (ITC). In this line, it was argued that this delay might be crucial to enable the integration of information from different cortical areas, giving rise to a unified concept (Quian Quiroga, 2012). A recent study has shown a global LFP deflection in the theta-band (4–8 Hz) that precedes the response onset of concept cells (**Figure 2**) and is present only when the stimulus is consciously recognized (Rey et al., 2014). Moreover, the precise onset of concept cell responses is characterized by an increase in phase locking between the spikes and the LFPs in the theta band.

Even though the neural origin of this LFP deflection remains unclear, we believe that it is not originated from within the MTL. This is partially accounted by the fact that the human hippocampus is not thought to produce substantial contributions to the low-frequency LFP signals due to its structure (Buzsaki et al., 2012). More importantly, since the theta activation was seen globally in the MTL, if it were generated within the MTL, single cell activity responsible for this should have been observed prior to the change in the LFP. However, this situation was not observed (Rey et al., 2014). In turn, we hypothesize that this LFP response may reflect an activation generated by reverberating activity in the ITC crossing a certain threshold and triggering perception. This LFP would provide a temporal window so that perceptual information can reach the MTL for further processes, such as memory functions.

## CONCLUDING REMARKS

This review discussed two methodological considerations in the study of the NCCs. In particular, we first argued for the implementation of paradigms where “seen” and “unseen” trials are obtained through the use of identical stimuli. Using contrastive analysis with identical visual stimulation allows ruling out physical effects that can otherwise contaminate the comparison between conditions. In the second part we discussed a selection of studies in which different NCCs were found at different timings and different brain areas (Quian Quiroga et al., 2008; Navajas et al., 2013; Rey et al., 2014). These seemingly contradictory results can be put together into a coherent framework by discriminating two different neural processes associated with conscious perception (i.e., perceptual and contextual awareness).

Interestingly, other distinctions between different conscious processes in the brain were previously proposed (Block, 1995; Kouider et al., 2010). For example, Block introduced the dichotomy between “phenomenal” and “access” consciousness (for a review, see Block, 2005), which is mainly centered on the question of whether we can have cognitive access to all our perceptual experiences, and thus whether we can see more than we can report (Block, 2011, 2012; Kouider et al., 2012). In this review, we proposed to discriminate between two different types of consciousness for already perceived stimuli (that can be reported) – i.e., processes beyond the distinction of phenomenal and access consciousness. In particular, we distinguish between a type of consciousness that relies on the firing of concept cells (*contextual awareness*) and the one that can be experienced even in the absence of MTL structures (*perceptual awareness*). The most remarkable examples of *perceptual* without *contextual* awareness are provided by patients with bilateral MTL resection or damage, such as patients H.M. (Scoville and Milner, 1957), R.B. (Zola-Morgan et al., 1986), and K.C. (Steinvorth et al., 2005). This condition led to a severe impairment in recollecting autobiographical events with no temporal gradient (Steinvorth et al., 2005), deficits in imagining new experiences (Hassabis et al., 2007), as well as in retaining and retrieving any type of episodic memory (Moscovitch et al., 2005). However, many other cognitive functions remained unaltered in these patients, such as the recognition of faces encoded before the surgery/accident (Kensinger and Corkin, 2000; O’Kane et al., 2004). Altogether, this evidence indicates that the MTL is not involved in the recognition of semantic entities, a process that we propose to be triggered by object-selective cortical areas (*perceptual awareness*). But bilateral damage or resection of MTL structures prevents subjects from having an enriched representation of the context, associations, and episodic memories linked to the specific stimulus, which we argue is elicited by the firing of concept cells (*contextual awareness*).

## Conflict of Interest Statement

The authors declare that the research was conducted in the absence of any commercial or financial relationships that could be construed as a potential conflict of interest.
